# Explicating the Pivotal Pathogenic, Diagnostic, and Therapeutic Biomarker Potentials of Myeloid-Derived Suppressor Cells in Glioblastoma

**DOI:** 10.1155/2020/8844313

**Published:** 2020-11-04

**Authors:** Seidu A. Richard

**Affiliations:** Department of Medicine, Princefield University, P. O. Box MA 128, Ho, Ghana

## Abstract

Glioblastoma (GBM) is a malignant and aggressive central nervous tumor that originates from astrocytes. These pathogenic astrocytes divide rapidly and are sustained by enormous network of blood vessels via which they receive requisite nutrients. It well proven that GBM microenvironment is extremely infiltrated by myeloid-derived suppressor cells (MDSCs). MDSCs are a heterogeneous cluster of immature myeloid progenitors. They are key mediates in immune suppression as well as sustenance glioma growth, invasion, vascularization, and upsurge of regulatory T cells via different molecules. MDSCs are often elevated in the peripheral blood of patients with GBM. MDSCs in the peripheral blood as well as those infiltrating the GBM microenvironment correlated with poor prognosis. Also, an upsurge in circulating MDSCs in the peripheral blood of patients with GBM was observed compared to benign and grade I/II glioma patients. GBM patients with good prognosis presented with reduced MDSCs as well as augmented dendritic cells. Almost all chemotherapeutic medication for GBM has shown no obvious improvement in overall survival in patients. Nevertheless, low-dose chemotherapies were capable of suppressing the levels of MDSCs in GBM as well as multiple tumor models with metastatic to the brain. Thus, MDSCs are potential diagnostic as well as therapeutic biomarkers for GBM patients.

## 1. Introduction

Glioblastoma (GBM) is a malignant and aggressive central nervous tumor that originates from astrocytes [1, 2]. Astrocytes are star-shaped cells and are recognized to maintain brain tissue [1]. In GBM, pathogenic astrocytes divide rapidly and are sustained by enormous network of blood vessels via which they receive requisite nutrients [1, 2]. Currently, standard management for patient with GBM comprises of maximal safe surgical resection, subsequent to concurrent radiation therapy as well as chemotherapy [3, 4]. The current chemotherapeutic medication includes temozolomide, lomustine, bevacizumab, and carmustine wafers [3, 5]. Nevertheless, none of these medications have shown obvious improvement in overall survival [3, 5]. The GBM microenvironment is extremely infiltrated by myeloid-derived suppressor cells (MDSCs) [1, 6–8].

MDSCs are a heterogeneous cluster of immature myeloid progenitors [9]. They are key mediates in immune suppression as well as sustenance glioma growth, invasion, vascularization, and upsurge of regulatory T cells via different molecules [9]. MDSCs are derived from monocytes and attain immunosuppressive ability during certain disease circumstances most especial cancer [10–12]. Human MDSCs are often depicted with pan-myeloid marker CD33, with monocytic CD14^+^ as well as granulocytic CD15^+^ subsets [10]. Interestingly, GBM microenvironment is usually depicted with the elevation of MDSC levels [10, 13]. Several studies have demonstrated that MDSCs are capable of blocking T cell as well as natural killer (NK) cell functions [14–16]. These blockades resulted in immunocompromise as well as a tumor-facilitatory microenvironment [14, 17, 18].

Studies have demonstrated that MDSCs via different mechanisms are capable of blocking T cell stimulation as well as expansion [11, 19, 20]. These mechanisms involve the generation of arginase and inducible nitric oxide synthase (iNOS), reactive oxygen species and/or reactive nitrogen species (ROS and/or RNS), secretion of interleukin- (IL-) 10, and upsurge in regulatory T cells (Tregs), as well as blockade of T cell migration [11, 19–21]. Furthermore, MDSCs were capable of facilitating immunosuppression as well as tumor succession [11, 19, 20]. In view of lack of concrete biomarkers for GBM, this review explores the fundamental pathogenic and diagnostic as well as therapeutic biomarker potentials of MDSCs in GBM. The “boolean logic” was utilized to search for article on the subject matter. Most of the articles were indexed in PubMed and PubMed Central with strict inclusion criteria being *in vitro* and *in vivo* up or downregulation as well as therapeutic potentials of MDSCs in GBM.

## 2. MDSC Types and Subtypes in Glioma

Granulocytic/polymorphonuclear (G-MDSCs/PMN-MDSCs) and monocytic (M-MDSCs) are two groups of MDSCs [22, 23]. These two groups of MDSCs are phenotypically as well as morphologically analogous to neutrophils and monocytes, correspondingly [22, 23]. Elevated levels of both groups have been detected in the blood of patients with GBM [24]. Nevertheless, elevated levels of M-MDSCs correlated with tumor grade [24]. Dubinski et al. observed a downregulation of CD16 and upregulation of HLA-DR in both G-MDSCs and M-MDSCs at the tumor microenvironment of GBM tissues [22]. Nevertheless, the downregulation of CD16 and upregulation of HLA-DR were more prominent in M-MDSCs compared to G-MDSCs [22]. On the other hand, while eosinophilic MDSCs showed a robust upregulation of HLA-DR, neutrophilic MDSCs exhibited a dissimilar HLA-DR secretory pattern [22].

MDSCs are further subgrouped into CD11b^+^CD45^low^ and CD11b^+^CD45^high^ based on CD45 secretory levels in both rodent and human GBM [14]. Furthermore, CD11b^+^CD45^low^ cells are believed to be microglia while CD11b^+^CD45^high^ cells are believed to be peripheral macrophages [14, 25]. Nevertheless, the CD11b^+^CD45^high^ cells comprise of both macrophages and microglia [14, 26]. Also, in rodent GBM models, CD11b^+^CD45^high^ cell fraction was the predominantly expressed when the samples were stimulated with proinflammatory and immunosuppressing molecules [14]. Therefore, the CD11b^+^CD45^high^ cells appear to be the most reactive cells in GBM microenvironment influencing tumor progression [14].

In GBM microenvironment, the CD11b^+^CD45^high^ cell fraction, comprising microglia as well as macrophages, increased in a time-dependent manner, and naïve microglia triggered upregulate of CD45 [14, 26]. Comparatively, the stimulation of molecules like major histocompatibility complex (MHC) classes I and II, GR1, CD80, and CD86, as well as IL-10 showed observable effects in CD11b^+^CD45^high^ cells, while in CD11b+CD45^low^ fraction, no observable effects were visible [14]. Severe studies have also implicated CD11b^+^GR1^+^ cells as MDSC subtypes in GBM [14, 27, 28]. This subtype was capable of blocking antitumor responses as well as accelerated tumor progression [14, 27, 28]. Brandenburg et al. observed a robust upregulation of GR1 in MDSCs obtained from glioma samples which was limited to only the CD11b+CD45^high^ population [14]. They indicated that the high secretion of GR1 in CD11b+CD45^high^ cells as well as their potential suppressive phenotype could inhibit some proinflammatory factors [14].

Studies have shown that tumor-bearing mice depleted of CD11b^+^Gr1^+^ cells survive longer compared to those treated with control isotype IgG [17, 29]. This means that this immune group actively participated in the pathogenesis as well as prognosis of gliomas [29]. CD11b^+^Gr1^+/high^ cells were the most predominate BIL population in the brain. CD11b^+^Gr1^low^ cell secretion resulted in an upregulation of IL-4R*α* in the tumor microenvironment as compared to healthy brain cells [29, 30]. MDSCs constituted about 5% of the cells within GBM tumor masses and where depicted with obvious CD33^+^/CD15^−^/CD14^−^/HLA-DR^−^ negative lineage subsequent to CD33^+^/CD15^+^/CD14^−^/HLA-DR^−^ neutrophilic as well as CD33^+^/CD15^−^/CD14^+^/HLA-DR^−^ monocytic subtypes [29–31].

## 3. MDSCs at the Glioblastoma Microenvironment

Several studies have demonstrated that MDSCs amass in the tumor and spleen, as well as the peripheral blood of patients with GBM [11, 13, 22, 28, 31–33]. They are capable of eliciting immune blockade via dampening the function of NK cells and cytotoxic T lymphocytes (CTLs) in these tissues [11, 13, 22, 28, 31–33]. Several studies have further demonstrated that MDSCs infiltrate the glioma microenvironment and function as drivers of immune-inhibitory phenotype distinctive to these lesions [10, 34, 35]. Suppression of MDSCs within glioma microenvironment facilitated survival as a result of concurrent upsurge of MDSCs within bone marrow, as well as an upsurge in functional tumor-infiltrating lymphocytes [34].

In the GBM microenvironment, angiogenin, insulin-like growth factor binding protein (IGFBP 2&3), IL-6, IL-8, monocyte chemoattractant protein-1 (MCP-1/CCL2), macrophage migration inhibitory factor (MIF), and osteopontin, as well as tissue inhibitor of metalloproteinases (TIMP 1&2) were significantly elevated [36]. Several studies have demonstrated that chemokine receptors particularly chemokine receptor 2 (CCR2) were capable of activating the collection of MDSCs at the GBM microenvironment [10, 34, 37]. Also, studies have demonstrated that GBM was capable of secreting IL-8 which in turn triggered the trafficking of MDSCs into the tumor environment via the CXCR2 receptor which functions as MIF receptors [36, 38]. Studies have further demonstrated that higher levels of MCP-1/CCL-2 are secreted GBMs as well as other glioma variants [36, 39, 40]. MCP-1/CCL-2 secreted by GBMs is capable of trafficking MDSCs into the tumor microenvironment and felicitated tumor growth via MDSCs [36, 39, 40].

In GBMs, monocytic Ly6C-secreting MDSCs are the principal constituent of the GBM microenvironment and represent about 40% of the total tumor mass [34, 37]. Ly6C^high^ inflammatory monocytes principally secret CCR2 [34, 37]. Flores-Toro et al. observed a reduction in CD11b^+^/Ly6C^high^/PD-L1^+^ MDSCs in GBMs which was associated with an upsurge in overall CCR2^+^ cells as well as MDSCs within bone marrow of CCR2-deficient mice [34]. They further established that CCR2 antagonist CCX872 prolonged median survival as a monotherapy in KR158 glioma-bearing animals [34]. Furthermore, it also prolonged median as well as overall survival when combined with anti-PD-1 [34]. Nevertheless, a combination of CCX872 and anti-PD-1 lengthened median survival time in 005 GSC GBM-bearing mice [34]. Also, in both models, CCX872 reduced tumor-related MDSCs as well as augmented these cells within the bone marrow [34].

NF-*κ*B signaling demonstrated to be capable of mediation in MDSC expansion in an aging brain model [41, 42]. Tumor-associated myeloid cells deficient in NF-*κ*B signaling exhibit anti-inflammatory properties, which was seen as augmentation in inflammatory mediators subsequent to restricted deletion of p65 in myeloid cells in GBM models [41]. On the other hand, Janus Kinase (JAK)/Signal Transducer and Activator of Transcription (STAT) pathway is often stimulated during inflammatory and immune responses [41–43]. This pathway has demonstrated to be capable of influencing MDSC reaction in response to several stimuli [41, 43].

NF-*κ*B was capable of triggering STAT3 resulting in the induction of indoleamine 2,3-dioxygenase (IDO) enzyme upregulation which functions as an immune suppressive mediator in MDSCs [41, 43]. Also, stimulation of the IDO enzyme in dendritic cells (DCs) resulted in GBM immune evasion [44, 45]. Furthermore, tryptophan was extremely metabolized in tumor microenvironment [44]. Also, tryptophan metabolisms which were initially observed in peripheral tolerance and maternal tolerance of fetus as well as stimulation of autoimmune disease were associated with MDSC immunosuppressive mechanisms [44, 45]. Also, IL-6 is an effective stimulator of STAT3 which has demonstrated to regulate MDSC-facilitated immunosuppression [36, 46].

MDSCs are capable of blocking adaptive immunity of the body via the triggering of Treg cells [1, 47]. Treg cells are CD4^+^ T cells with associated prominent FOXP3 and are recognized to be capable of inhibiting innate immune responses [1, 48]. Arginase-1 (Arg-1) was capable of triggering MDSC immunosuppression via the depletion L-arginine fundamental for growth as well as differentiation of T cells, leading to T cell dysfunction [9, 49]. Huang et al. demonstrated that MDSCs were capable of stimulating the development of Treg cells both *in vitro* and *in vivo*, via the stimulation of TGF-*β* and IL-10 as well as cell-cell communication [50]. MDSC modifications resulted in a reduction in TCR binding to MHC I-peptide complexes in CD8^+^ T cells as well as decreased the ability of these cells to respond to antigen peptides [44].

MDSCs generated excessive ROS as well as RNS which were capable of inhibiting the maturation of DC which in turn resulted in the accumulation of MDSCs [44]. Also, ROS and RNS have been implicated in T cell inactivation. Furthermore, peroxynitrite generation resulted in posttranslation protein alterations via the nitration of tyrosine, cysteine, and methionine as well as tryptophan [44]. MDSCs are capable of inhibiting the production and storage of T cells in lymph nodes [44]. Naive T cells infiltrate secondary lymph nodes via the secretion of L-selectin and become stimulated via antigen peptides originating from tumor sites through lymphatic vessels [44, 51]. MDSCs are capable of downregulating L-selectin secretion on naive T cell surface via the release of ADAM metallopeptidase domain 17 (ADAM17, 44, and 51). ADAM17 is associated with the proteolytic cleavage and shedding of the L-selectin ectodomain [44, 51].

Freshly isolated blood-derived neutrophilic and less efficiently eosinophilic MDSCs were capable of inhibiting autologous nonspecific T cell proliferation and IFN-*γ* secretion *in vitro* and not monocytic MDSCs [22]. Studies have shown that peripheral cellular immunosuppression in patients with GBM was mediated by degranulated neutrophils or CD33^+^HLA^−^DR^−^CD15^+^ cells [13, 22, 52]. Tumor-related MDSCs secreted higher levels of MHC-II compared to microglia and macrophages in patients with GBM [53]. Contrary, MDSCs were characterized by deficiency of MHC-II secretion and the existence of the myeloid marker CD33 in blood or CD11b in tumor tissue [33].

Kohanbash et al. demonstrated that IL-4R*α* was upregulated in myeloid cells precisely at the tumor microenvironment but not in the periphery in patients with GBM and *de novo* murine glioma models [30]. They further revealed that GM-CSF, which is distinctively upregulated in the glioma microenvironment, triggered the secretion of IL-4R*α* in myeloid cells resulting in the promotion of IL-13-stimulated Arg-1 secretion as well as T cell suppression *in vitro* cultured cells as well as cells isolated from glioma-bearing hosts [30]. Future studies should focus on the functional role of MDSCs in gliomagenesis as well as malignant degeneration.

## 4. TAM and Glioblastoma

In GBMs, tumor-associated macrophages (TAMs) constitute about 20-30% of the cells in the entire tumor [54, 55]. TAMs are capable of secreting soluble factors as well as surface molecules that inhibit immune surveillance via endogenous T cells and NK cells resulting in the blockade of crosstalk between the adaptive and innate immune systems [54, 56, 57]. TAMs are classified into M1 macrophages and M2 macrophages [54, 55]. Several studies have demonstrated that GBMs are depicted cardinally with the infiltration of TAM which influences the tumor progression, with both resident microglia and bone marrow-derived infiltrating monocytes capable of eliciting tumor growth-facilitatory activities [1, 6–8]. In GBM, fraction of TAM infiltration of the tumor correlated positively with the tumor grade [9, 58].

Classically stimulated M1 macrophages trigger antitumor response via generation of proinflammatory cytokines, presenting antigens to adaptive immune cells as well as phagocytosing tumor cells [9]. MDSCs are capable of secreting markers for proinflammatory M1 cells like iNOS, IL-1*β*, and TNF-*α*, as well as CXCL10 [29]. Also, MDSCs are capable of secreting tumor-supportive M2-polarized macrophages like Arg-1, CCL17, and CD206, as well as CD36 [29]. Thus, MDSCs are pleiotropic nature [29]. Conventionally, studies have demonstrated that the alternatively secreted M2 macrophages secret immunosuppressive cytokines like STAT3 as well as scavenger receptors like CD163, CD204, and CD206 and facilitate tumor supportive CD4^+^ regulatory T cells [9, 59].

Studies have demonstrated that TAMs and MDSCs often form a sizeable percentage of tumor-infiltrating immune cells in the GBM tumor microenvironment [23, 60–62]. They range from 30-90% in human GBM samples, with CD11b^+^ MDSCs constituting the majority of infiltrating inflammatory cells in human gliomas [23, 61, 62]. Microglial TAMs were enhanced in the principal edge of the tumor as well as surrounding white matter in a study involving single-cell RNA sequencing of gliomas while blood-derived TAMs were more often seen within regions of microvascular proliferation as well as perinecrotic regions within the core of the tumor [23]. This is correlated with intense secretion of proinflammatory factors in the periphery as well as anti-inflammatory factors in the core [23, 63].

Also, TAMs that arose from the blood and migrate to brain tumors, where they assume an additional tissue-specific phenotype, proved to have a different metabolism and augmented secretion of immunosuppressive markers compared to microglia [23, 64]. Moreover, as glioma grade ascended, the proportion of blood-derived TAMs to microglia simultaneously ascended too [23, 65]. Nevertheless, notwithstanding the augmented affinity of microglia toward a proinflammatory phenotype, both cell categories have the possibility of triggering tumor-based microenvironment toward MDSCs and therefore are potential targets for myeloid therapy [23, 66].

## 5. MDSCs and Macrophage Migration Inhibitory Factor

Macrophage migration inhibitory factor (MIF) is derived from GBM cells, precisely medicinally resistant cancer stem cells, and it is obligatory for MDSC survival and function [32, 67]. Downregulation of MIF levels in GBM cells was incapable of modifying their proliferation [32, 67]. Nevertheless, an augmented host survival and an upsurge in the quantity of CD8^+^ T cells in the tumor microenvironment were observed when they were transplanted into an immune-proficient orthotopic model [32, 67, 68]. Also, MDSCs are capable of influencing macrophages resulting in inflammation as well as immunosuppressive effects in the tumor microenvironment [1, 69].

GBM tumors are often depicted with immunosuppressive milieu as well as participate in numerous pathways leading evasion of innate immune surveillance [1]. Most importantly, macrophage subtypes, principally M1 and M2, are the main origins of inflammatory cytokines [1]. M1 macrophages express IL-12 that facilitates the production of T helper 1 (Th1) adaptive immunity resulting in the triggering of cytotoxic effects in tumor cells [1, 70]. On the other hand, M2 macrophages are immune suppressive cells which are capable of facilitating matrix remodeling leading to tumor progression [1, 71]. M1 macrophages are depicted with elevated secretion of CD86 as well as IL-12 [1, 72].

A crosstalk between MDSCs and GBM tumor stem cells via MIF resulted in an augmented MDSC function as well as an upsurge in cytotoxic T cell infiltration, which may be a potential target for inhibiting GBM progression [35, 67]. Kumar et al. established that MIF was prominent across all three of their glioma prepared samples [36]. Studies have shown that MIF was significantly secreted by GBM cells resulting GBM-mediated immunosuppression [36, 73, 74]. The most crucial MIF inhibitor is the 4-iodo-6 phenylpyrmidine (4-IPP) which has also demonstrated to be capable of inhibiting MDSC development [36, 75, 76].

## 6. MDSCs and Glioblastoma Hypoxia

Hypoxia is very fundamental in the progression as well as immunosuppressive function of GBMs. Hypoxia-triggered glioma culture medium was capable of differentiating tumor-associated macrophages to immunosuppressive M2 subtype [77, 78]. In hypoxic milieu, GBM cells are induced to express distinctive kinds of exosomes that are transported to normoxic zones of the tumor to facilitate the tumor progression [77, 79]. Exosomes are tiny vesicles of about 50-150 nm in diameter present in all bodily fluids and are expressed by both normal and malignant cells [77, 80]. Several studies have demonstrated that tumor exosomes transport genetic materials as well as proteins efficient in inhibiting the functions of immune cells as well as stimulating the triggering and expansion of MDSCs *in vitro* and *in vivo* [77, 81].

Several studies have demonstrated that hypoxia was capable of influencing the secretion and quantities of miRNA by exosomes which resulted in MDSC differentiation and accumulation [77, 79, 82]. Studies have further shown that GBM-derived exosomes (GDEs) are able to modify the mRNA secretory profiles of their fibroblasts as well as TAMs [77, 83, 84]. Guo et al. demonstrated that hypoxia facilitated the secretion of exosomes via GBM and that normoxia-stimulated GDEs (N-GDEs) as well as hypoxia-stimulated (H-GDEs) were taken up by MDSCs in mice [77]. They indicated that H-GDEs were more capable of triggering the expansion as well as immunosuppressive activities of MDSCs *in vivo* and *in vitro* [77]. Furthermore, miR-10a and miR-21 were upregulated in H-GDEs and had the most robust MDSC stimulatory influence amongst the 20 highest secretory GDE miRNAs [77].

Hypoxia-stimulatory miR-10a as well as miR-21 secretion in GDEs augmented the expansion as well as suppressive actions of MDSCs [78]. Thus, miRNAs are capable of being transferred from tumor cells to MDSCs via GDE crosstalk [77]. Extracellular transfer of hypoxia-stimulatory miR-10a as well as miR-21 in GDEs led to MDSC stimulation and downregulation of miR-10a as well as miR-21 target proteins [77, 78]. Hypoxia facilitated the expression as well as upregulation of miR-10a and miR-21 levels in GDEs [77]. Furthermore, H-GDEs miR-10a and miR-21 facilitated MDSC stimulation via the Rora/I*κ*B*α*/NF-*κ*B and Pten/PI3K/AKT pathways [77, 78]. Also, reduced MDSCs were detected in the spleens of mice bearing miR-10a or miR-21 knockout glioma cells compared to those bearing normal glioma cells [77]. Studies have proven that hypoxia-stimulated upregulation of miR-155 and miR-584 and the downregulation of miR-244 were associated with glioma progression [77, 85–87].

## 7. MDSC and T Cells

GBMs are highly immunosuppressive brain tumors that are well recognized for their T cell paucity [88]. GBMs are capable of escaping immunosurveillance via the trapping of T cells in the bone marrow via the loss of sphingosine-1-phosphate (S1P) receptor on the T cell surface [88]. Thus, GBMs were capable of escaping immunosurveillance via the locking away T cells in the bone marrow [88]. Bone marrow aspirates from treatment-naive GBM patients as well as GBM-bearing mice showed extreme T cells in the bone marrow compared to the blood, while controls had matching T cell quantities in both bone marrow and blood [88]. Also, T cells were also scanty in the spleen, resulting in splenic shrinkage which implied that T cell generation in the bone marrow may be shrunken [88].

Domenis et al. observed a blockade of CD4^+^ T cell effector activities in unfractioned peripheral blood mononuclear cells (PBMCs) [89]. They explained that this blockade effect was as a result of the stimulation of monocytes, which required the skewing of immature immunophenotype towards the expansion of M-MDSCs and not as a result of direct delivery of exosomes to T cells [89]. They further indicated that GDEs were associated with the conversion of monocytes to Arg-1- and IL-10-generating M-MDSC cells that resulted in T cell immunosuppression without obligatory direct contact between monocytes and GBM cells [89].

MDSCs were capable of suppressing T cell activities via numerous mechanisms, including the generation as well as expression of nitric oxide (NO), Arg-1, and ROS most especially H_2_O_2_ [22, 33, 90]. Also, MDSCs' ability to suppress T cell responses resulted in the depletion of specific amino acids like L-arginine which are fundamental for T cell function. Arg-1 and nitric oxide synthase 2 (NOS2) are the two main catabolic enzymes via which MDSCs metabolize L-arginine [11, 22]. Patients with freshly detected GBM had an augmented quantity of circulating CD33^+^ HLA-DR2 MDSCs in their blood which comprises of neutrophilic (CD15^+^) and immature (CD152 and CD142), as well as monocytic (CD14^+^) subsets [22].

On the other hand, knockout of MDSCs from PBMCs with anti-CD33/CD15-coated beads *in vitro* partly restored T cell generation of IFN-*γ* after induction with anti-CD3/anti-CD28 antibodies [22]. Moreover, MDSC was not restricted to only the suppressing of CD4^+^ and CD8^+^ T cells, but also suppressing of cytotoxic activities of NK and NK T cells [91]. Thus, during cell cycle, MDSC uses numerous mechanisms to suppress immune responses. These suppressive mechanisms include arresting T cells in the G0-G1 phase, interfering with T cell trafficking such as CD8^+^ T cells unresponsive to antigen-specific activation and stimulation as well as expansion of Treg cells, and stimulating energy generation in NK cells, suppressing NK cell cytotoxicity as well as potential stimulation of NK T cells [91].

## 8. MDSCs and B Cells

B cells are produced from lymphoid precursors in the bone marrow in a strictly regulated process, with stepwise recombination of V, (D) and J gene fragments coding for the variable (V) region of the immunoglobulin (Ig) heavy as well as the light chains [92, 93]. B cells are capable of producing functional, nonautoreactive B cell receptor (BCR) which differentiates into mature, naive B cells [92]. B cells are capable of proliferating as well as differentiating into plasma cells without the support of T cells when B cell receptors are stimulated [92]. B cells are stimulated in the periphery before they migrate into secondary lymphoid tissues [92]. Furthermore, another route which B cells are stimulated locally via floating antigen or immune complexes was presented by DCs [92].

B cells have demonstrated to fundamental in the GBM immune landscape [94, 95]. Lee-Chang et al. demonstrated that human as well as murine GBM-associated B cells exhibit an immunosuppressive phenotype depicted with the presence of inhibitory molecules such as PD-L1 and CD155 as well as the production of immunoregulatory cytokines like TGF-*β* and IL-10 [94]. They observed that regulatory B cells (Bregs) constituted about 10% of bone marrow-derived infiltrating immune cells in GL261 and CT2A orthotopic brain tumor models, and 40% of GBM patients were positive for B cell tumor infiltration during screening [94].

GBM-associated B cells exhibited immunosuppressive activities toward stimulated CD8^+^ T cells, and their pathophysiological significance was underlined by prolonged animal survival after local administration of B cell-depleting immunotherapy [94]. At the tumor microenvironment, MDSCs were capable of facilitating immunosuppression of B cells [94]. Also, MDSCs facilitated the transfer of membrane-bound PD-L1 to B cells, leading to the facilitation of B cell-associated immunosuppression [94]. Lee-Chang et al. detected that local intratumoral depletion of B cells correlated well with the enhancement of animal survival [94].

Moreover, the therapeutic efficiency of depleted of B cells was associated with augmented intratumor GzmB^+^ CD8^+^ T cells [96, 97]. This means that depletion of GBM-associated Bregs promoted CD8^+^ T cell stimulation and facilitated their effector function [96, 97]. On the other hand, *ex vivo* tumor-infiltrating B cells from GBM tumor samples demonstrated immunosuppressive activities toward CD8^+^ T cell stimulation [96, 97]. Also, adoptive transfer of naïve B cells not only salvaged the survival phenotype but also demonstrated that only tumor-infiltrating B cells exhibit elevated PD-L1 and CD155 [94]. Thus, dynamic process most likely occurred in the tumor microenvironment that switches naïve B cells into GBM-associated Bregs [94].

## 9. MDSCs and Interferons

Interferons (IFNs) are effective and extensively active cytokines that are capable of triggering cellular responses to nucleic acids of abnormal structure or location [98, 99]. Interferons have been detected in normal as well as malignant cells of all lineages in response to pathogenic molecular stimuli [98]. Interferons were capable of triggering transcription of numerous genes resulting in protein synthesis in both cellular and viral, autophagy, apoptosis, and angiogenesis as well as innate and adaptive immunity [98, 99].

MDSCs are able to illicit their immunosuppressive functions via feedback reaction to IFN-*γ* release by stimulated T cells, which in turn triggers the expression of IL-10 and TGF-*β* [50, 100]. The suppressive functions of MDSC was associated with IFN-*γ* secretion because MDSCs from IFN-*γ*^−/−^ mice did not have the above capabilities [100, 101]. The presence of antibodies against mouse IFN-*γ* did not result in the elevation of lytic response in normal allo-cultures lacking MDSCs, while it does restore entirely immune responsiveness in cultures containing MDSCs [101]. Furthermore, exogenous IFN-*γ* was capable of replacing cytokines generated by either T lymphocytes or MDSCs derived from either the spleen or tumor infiltrate in supporting T lymphocyte blockade [101].

Pituch et al. demonstrated that, analogous to human chimeric antigen receptor (CAR) T cells, murine CAR T cells were associated with IL13R*α*2-secreting glioma cells [102]. This was observed via their cytolytic actions as well as the production of IFN-*γ* and TNF-*α* in the presence of IL13R*α*2-positive glioma cells [102]. They indicated that IL13R*α*2-CAR T cells had antiglioma actions in two syngeneic glioma models and produced a proinflammatory tumor microenvironment in their *in vivo* experiments [102]. IL13Ra2-CAR.CD28.*ζ* T cell proliferation resulted in the production of IFN-*γ* and TNF-*α* as well as facilitated phenotypically proinflammatory glioma microenvironment by triggering elevated levels CD4^+^ and CD8^+^ T cells and CD8*α*^+^ DCs as well as a reduction in Ly6G^+^ MDSC levels [102].

IFN-*γ* was capable of binding with receptors that resulted in the stimulation JAK/STAT signaling pathway, which in turn resulted in the downregulatory secretion and stimulation of IRF-1, leading to secretion of PD-L1 by tumor cells [103, 104]. Studies have shown that, PD-1/PD-L1 was capable of reversing the immune evasion of glioma [103, 105]. Nduom et al. observed that PD-L1 was a negative prognostic indicator for GBM during their study involving 94 GBM patients [106].

Qian et al. found that the distribution of PD-L1 in glioma corresponded with morphologically apoptotic T cells [103]. They further observed that IFN-*γ* triggered PD-L1 release in primary cultured microglia and bone marrow-derived macrophages as well as GL261 tumor cells [103]. They concluded that IFN-*γ* derived from tumor-infiltrating T cells was capable of triggering PD-L1 secretion in the tumor microenvironment [103]. Studies on IFN-*γ*/PD-L1/MDSC axis are warranted.

## 10. MDSCs in Glioblastoma Therapy

**Figure 1:**
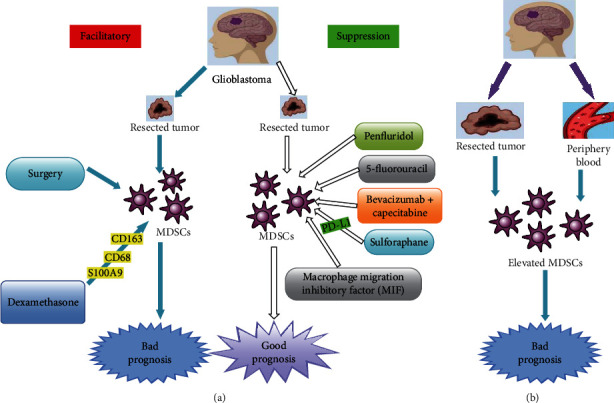
Diagnostic and therapeutic biomarker potentials of myeloid-derived suppressor cells in glioblastoma. (a) Shows therapeutic biomarker potential of MDSCs after various chemotherapeutic agents as well as surgical outcomes. Facilitation of MDSC secretion leads to bad prognosis while suppression of MDSCs leads to good prognosis. (b) Shows the diagnostic biomarker potentials of MDSCs. MDSCs are elevated in both glioblastoma tumor and the periphery blood indicating bad prognosis.

GBMs which are exceedingly heterogeneous, comprising of tumor cells, tumor-activating cells, infiltrating immune cells, and endothelial cells as well as other tumor-related stromal cells are often observe at cellular level which makes developing targeted therapies very difficult [1, 6–8]. Studies have demonstrated that MDSCs may contribute to the failure of immune therapies for GBM because they are capable of potentiating immune suppression in GBM patients [10, 34, 37]. Ranjan et al. demonstrated that oral administration of 10 mg/kg penfluridol daily suppressed the growth of GBM tumors in a subcutaneous as well as intracranial model [1, 107]. They indicated that oral administration of 10 mg/kg penfluridol daily for 48 days also reduced mouse MDSCs by 72% (Figure 1(a)) [1]. They further demonstrated that penfluridol treatment drastically suppressed the growth of metastatic breast tumors in brain [1, 108].

Several studies established that low-dose chemotherapies were capable of suppressing the levels of MDSCs in multiple tumor models [3, 109, 110]. Peereboom et al. demonstrated that 5-fluorouracil (5-FU) was capable of suppressing MDSCs in preclinical GBM mouse models (Figure 1(a)) [3]. 5-FU is an antimetabolite drug that enters both RNA and DNA via the blockade of thymidylate synthase resulting in decrease dTTP concentrations [3]. It was established that dUTP and FdUTP have less competition and are thus more likely to enter DNA [3, 67, 111]. Metronomic low-dose 5-FU was capable of suppressing circulating MDSCs and augmented intratumoral-stimulated T cells as well as prolonged survival (Figure 1(a)) [3, 67].

Current treatment of GBM involves the use of corticosteroids prior to surgery [35]. A new trend points to the fact that steroids are capable of modifying MDSC subtypes in GBM patients toward the augmentation of immune suppression [35]. This strengthens the idea of targeting MDSCs in GBM patients, because all receive steroids therapy at the time of surgery [35]. Alban et al. also observed immunosuppression intratumorally, where MDSCs correlated with good prognosis of patients with GBM (Figure 1(b)) [35]. They used matched primary as well as recurrent tumor-resection samples to arrive at the above [35]. They further observed elevated levels of CD33^+^ MDSC levels at recurrence correlated with overall survival, whereas infiltration of MDSCs into the tumor microenvironment correlated with poor prognosis (Figure 1(b)) [35].

Nevertheless, some patients may receive dexamethasone for several days to weeks before surgery because of symptoms resulting from extensive edema [24, 112]. Studies demonstrated that the extent of dexamethasone therapy correlated with an upsurge in MDSCs in blood of glioma patients, as well as in experimental glioma mice models (Figure 1(a)) [24, 113]. Gielen et al. did not observe any correlation between dexamethasone and upsurge of MDSC within tumor tissues (Figure 1(a)) [24]. Also, a similar study did not find an effect of the duration of dexamethasone therapy on S100A8/9 or arginase secretion [24]. Further studies on the effects of dexamethasone on MDSCs in GBM patients are needed because the current evidences are conflicting.

Several studies observed a spike upsurge in MDSC levels in peripheral blood immediately after their surgical resections in primary GBM patients as well as recurrent GBM patients put on trial chemotherapeutic agents (Figure 1(a)) [3, 35, 114]. Also, elevated levels of MDSCs in the peripheral blood after surgery in GBM patients were observed after surgical intervention in multiple cancer types as well as in untreated GBM patients [3, 115–117]. Peereboom et al. indicated that their tumor immune profiles were analyzed on surgical samples obtained before bevacizumab therapy although all the patients on their trial received both capecitabine and bevacizumab [3]. Thus, the observed intratumoral consequences of capecitabine were not triggered by bevacizumab therapy [3].

Moreover, MDSC modifications began to appear before the beginning of bevacizumab although circulating MDSC levels were analyzed while patients were on bevacizumab and capecitabine (Figure 1(a)) [3]. The diminishing MDSC levels also happened in a capecitabine dose-dependent manner (Figure 1(a)), making it unlikely that bevacizumab played a substantial role [3]. Nevertheless, the potential adjuvant properties of bevacizumab on circulating immune types could not be deduced from their results [3].

Moyes et al. evaluated the incidence of usual myeloid markers like CD163, CD68, and CD33, as well as S100A9 using quantitatively analyzed tissue microarrays made of samples taken from grades I-III astrocytoma's and GBM [54]. They found that CD163 and CD68, as well as S100A9 rates were elevated in dexamethasone-treated grade I astrocytoma and GBM compared to normal brain tissue and grades II and III tumors [54]. Dexamethasone is a resilient glucocorticosteroid drug with anti-inflammatory as well as immunosuppressant properties [54]. It is administered to brain-tumor patients with edema one day prior to surgery in order to decrease the edema before surgery and after surgery, and its usage is rapidly tapered [24, 112].

MIF has demonstrated to be an appealing therapeutic target to reverse glioma-mediated MDSC buildup (Figure 1(a)) [32, 36]. Kumar et al. observed that administration of anti-MIF-inhibiting antibody inhibited the production of CD14^+^/HLA-DR-MDSCs stimulated by glioma-conditioned media (GCM) in their experimental models [36]. Furthermore, GCM had intrinsic tautomerase function compatible with the presence of MIF that was suppressed by the administration of sulforaphane (SFN) [36]. SFN is nontoxic to leukocytes at moderate to high doses but exhibits some intrinsic antiglioma activities at lower doses [36]. Moreover, SFN at reasonably low doses decreased MDSC production in GCM [36].

GBM expressed relatively high levels of PD-L1 in M-MDSCs which was reduced upon administration of SFN [36]. Furthermore, SFN facilitated the development of proinflammatory DCs from monocytes cultured in both fresh media and GCM in addition to its effects in reducing MDSC levels [36]. Nevertheless, only mature DCs (CD83^+^) were observed in the presence of GCM [36]. Moreover, in addition to its proinflammatory properties, SFN was also directly toxic to glioma cells signifying that it might have extra therapeutic benefits in patients with GBM [36].

## 11. MDSCs as Biomarkers for Glioblastoma

In a study involving 481 lower-grade glioma patients, Jacobs et al. observed that rs147960238, which was situated in the tenth intron of CD163, and rs17138945, which was situated in the second intron of MET, were considerably correlated with survival [118]. Several studies have demonstrated that CD163 is a hemoglobin/haptoglobin complex receptor that is secreted by macrophages and microglia. It was established that CD163 was capable of influencing inflammation via macrophages [118–120]. On the other hand, MET is a receptor tyrosine kinase as well as a protooncogene that is often associated with the expansion of MDSCs [118, 121]. Studies have proven that MDSCs are often elevated in the peripheral blood of patients with GBM (Figure 1(b)) [34, 35]. Studies have further shown that MDSCs in the peripheral blood as well as those infiltrating the GBM microenvironment correlated with poor prognosis (Figure 1(b)) [24, 32, 35].

Several studies have demonstrated elevation of CD33+/HLA-DR-MDSCs in the peripheral blood of patients with GBM compared to normal controls (Figure 1(b)) [13, 29, 100]. The MDSCs observed in the blood of patients with GBM consisted mainly of CD33^+^/CD15^+^/CD14^−^/HLA-DR^−^ neutrophilic subset with very few CD33+/CD15−/CD14−/HLA-DR^−^ negative lineage as well as CD33^+^/CD15^−^/CD14^+^/HLA-DR^−^ monocytic subtypes [13, 29]. Moreover, studies identified an upsurge in circulating M-MDSCs in the peripheral blood of patients with GBM compared to benign and grade I/II glioma patients (Figure 1(b)) [10, 24, 32]. Furthermore, there was a correlation between the secretory levels of intracellular S100A8/9 and upsurge in M-MDSCs in low-grade glioma patients [24]. Also, serum levels of S100A8/9 correlated with the function of arginase [24]. However, the values observed in this study were not predictable for the percentage of intratumoral MDSCs [24].

Studies have shown that elevation of peripheral MDSC levels correlated well with their immunosuppressive phenotypes, as well as with tumors that were refractory to immune-stimulating therapies like immune checkpoint blockers in multiple solid-tumor models as well as clinical trials (Figure 1(b)) [3, 122, 123]. Alban et al. demonstrated that GBM patients with good prognosis presented with reduced MDSCs as well as augmented DCs [35]. Thus, MDSC differentiation may be linked to an upsurge in immune stimulation leading to a decrease in GBM growth [35]. They found that immunosuppressive MDSC levels were high in high-grade glial malignancies as well as in nonglial malignancies with brain metastases, whereas suppressive T cell subtypes were not augmented, as initially described [33, 35, 124, 125]. They also found that steroid use inclines toward being a substantial predictor of MDSC levels in a univariable linear model [35].

Chae et al. demonstrated that mice receiving tumor cells and monocytes concurrently had augmented intratumoral MDSCs at sacrifice contrary to mice receiving tumor cells alone [28]. They indicated that mice receiving tumor cells mixed with monocytes had twice fold the quantity of splenic as well as bone marrow MDSCs contrary to mice receiving only tumor cells or only monocytes or control mice deprived of intracranial injection [28]. Their study concluded that augmenting glioma-associated monocytes led to an upsurge in intratumoral as well as systemic MDSCs in their experimental model (Figure 1(b)) [28]. In another study, they observed that normal human monocytes developed MDSC-like phenotype upon interaction with glioma cells which means that augmented glioma-associated monocytes/macrophages were capable of augmenting systemic MDSCs in patients with GBM as well [100].

Dubinski et al. observed that in peripheral blood, both the fraction of CD14^high^CD15^pos^ M-MDSCs and CD14^low^CD15^pos^ G-MDSCs were elevated compared with healthy controls (Figure 1(b)) [22]. They further observed that majority of G-MDSCs comprised of CD14^low^CD15^pos^ neutrophilic MDSCs [22]. Nevertheless, at the tumor side, they observed that a large percentage of CD14^low^CD15^pos^ G-MDSCs did not only compose of neutrophilic CD14^low^CD15^high^ MDSCs but also elevated levels of CD14^high^CD15^pos^ M-MDSCs [22]. Raychaudhuri et al. also observed elevated levels of CD15^+^CD14^−^ G-MDSCs over CD15^−^CD14^+^ M-MDSCs in tumor specimens of GBM patients (Figure 1(b)) [31].

Gielen et al. practically entirely observed CD15^+^CD14^−^ MDSCs within GBM tissue located both in viable and necrotic tumor zones (Figure 1(b)) [33]. Soler et al. designed a novel differential diagnostic approach merging analysis of secretion of two MDSC biomarkers; traditional HLA-DR as well as novel vascular noninflammatory molecule 2 (VNN2^+^) secretion on CD14^+^ monocytes was obtained from PBMC of GBM and/or radiation necrosis (RN) patients [126]. They indicated that this novel liquid biopsy could eliminate the necessity for biopsy to differentiate GBM from RN utilizing a minimally invasive and cheap as well as safe technique [126].

## 12. Conclusion

MDSCs are often elevated in the peripheral blood of patients with GBM. MDSCs in the peripheral blood as well as those infiltrating the GBM microenvironment correlated with poor prognosis. CD33+/HLA-DR-MDSCs were elevated in the peripheral blood of patients with GBM compared to normal controls. Also, an upsurge in circulating M-MDSCs in the peripheral blood of patients with GBM was observed compared to benign and grade I/II glioma patients. Furthermore, there was a correlation between the secretory levels of intracellular S100A8/9 as well as an upsurge in M-MDSCs in low-grade glioma patients. Also, serum levels of S100A8/9 correlated with the function of arginase. GBM patients with good prognosis presented with reduced MDSCs as well as augmented DCs. Nevertheless, augmenting glioma-associated monocytes led to an upsurge in intratumoral as well as systemic MDSCs in experimental model. MDSCs may contribute to the failure of immune therapies for GBM because they are capable of potentiating immune suppression in GBM patients. Low-dose chemotherapies were capable of suppressing the levels of MDSCs in multiple tumor models. These observations point to the fact that MDSCs are potential diagnostic as well as therapeutic biomarkers for GBM patients.

## Abbreviations

Arg-1: Arginase-1
ADAM17: ADAM metallopeptidase domain 17
BCR: B cell receptor
CTLs: Cytotoxic T lymphocytes
CCR: Chemokine receptor
DCs: Dendritic cells
GBM: Glioblastoma
GDEs: GBM-derived exosomes
N-GDEs: Normoxia-stimulated GDEs
H-GDEs: Hypoxia-stimulated
G-MDSCs: Granulocytic MDSCs
GCM: Glioma-conditioned media
iNOS: Inducible nitric oxide synthase
IL: Interleukin
IGFBP: Insulin-like growth factor binding protein
IDO: Indoleamine 2,3-dioxygenase
IFNs: Interferons
JAK: Janus kinase
MDSCs: Myeloid-derived suppressor cells
M-MDSCs: Monocytic MDSCs
MCP-1: Monocyte chemoattractant protein-1
MIF: Macrophage migration inhibitory factor
NK: Natural killer
NO: Nitric oxide
NOS2: Nitric oxide synthase 2
PMN-MDSCs: Polymorphonuclear MDSCs
PBMCs: Peripheral blood mononuclear cells
ROS: Reactive oxygen species
RNS: Reactive nitrogen species
Tregs: Regulatory T cells
RN: Radiation necrosis
Bregs: Regulatory B cells
TIMP: Tissue inhibitor of metalloproteinases
TAMs: Tumor-associated macrophages
Th1: T helper 1
STAT: Signal transducer and activator of transcription
SFN: Sulforaphane
VNN2^+^: Vascular noninflammatory molecule 2
4-IPP: 4-Iodo-6 phenylpyrmidine
5-FU: 5-Fluorouracil.
